# On the feasibility of simple brain-computer interface systems for enabling children with severe physical disabilities to explore independent movement

**DOI:** 10.3389/fnhum.2022.1007199

**Published:** 2022-10-21

**Authors:** Erica D. Floreani, Danette Rowley, Dion Kelly, Eli Kinney-Lang, Adam Kirton

**Affiliations:** ^1^Department of Pediatrics, Cumming School of Medicine, University of Calgary, Calgary, AB, Canada; ^2^Alberta Children’s Hospital, Alberta Health Services, Calgary, AB, Canada

**Keywords:** brain-computer interface, power mobility, pediatrics, cerebral palsy, alternate access technologies

## Abstract

**Introduction:**

Children with severe physical disabilities are denied their fundamental right to move, restricting their development, independence, and participation in life. Brain-computer interfaces (BCIs) could enable children with complex physical needs to access power mobility (PM) devices, which could help them move safely and independently. BCIs have been studied for PM control for adults but remain unexamined in children. In this study, we explored the feasibility of BCI-enabled PM control for children with severe physical disabilities, assessing BCI performance, standard PM skills and tolerability of BCI.

**Materials and methods:**

Patient-oriented pilot trial. Eight children with quadriplegic cerebral palsy attended two sessions where they used a simple, commercial-grade BCI system to activate a PM trainer device. Performance was assessed through controlled activation trials (holding the PM device still or activating it upon verbal and visual cueing), and basic PM skills (driving time, number of activations, stopping) were assessed through distance trials. Setup and calibration times, headset tolerability, workload, and patient/caregiver experience were also evaluated.

**Results:**

All participants completed the study with favorable tolerability and no serious adverse events or technological challenges. Average control accuracy was 78.3 ± 12.1%, participants were more reliably able to activate (95.7 ± 11.3%) the device than hold still (62.1 ± 23.7%). Positive trends were observed between performance and prior BCI experience and age. Participants were able to drive the PM device continuously an average of 1.5 meters for 3.0 s. They were able to stop at a target 53.1 ± 23.3% of the time, with significant variability. Participants tolerated the headset well, experienced mild-to-moderate workload and setup/calibration times were found to be practical. Participants were proud of their performance and both participants and families were eager to participate in future power mobility sessions.

**Discussion:**

BCI-enabled PM access appears feasible in disabled children based on evaluations of performance, tolerability, workload, and setup/calibration. Performance was comparable to existing pediatric BCI literature and surpasses established cut-off thresholds (70%) of “effective” BCI use. Participants exhibited PM skills that would categorize them as “emerging operational learners.” Continued exploration of BCI-enabled PM for children with severe physical disabilities is justified.

## Introduction

The onset of independent locomotion is a precursor to a host of developmental changes in children in areas such as cognition, perception, communication, language, and social participation (Campos et al., 2004; Anderson et al., 2013; Leonard and Hill, 2014). Children with severe motor disabilities, such as quadriplegic cerebral palsy, are limited in their ability to mobilize independently and are at risk for many secondary impairments. Immobility further denies these children their fundamental rights to participation and inclusion. Such barriers can lead to reduced motivation, engagement, self-confidence, and quality of life compared to their typically developing peers (Longo et al., 2013; Livingstone and Field, 2015).

Children with severe motor disabilities could benefit greatly from access to power mobility devices (PMDs), which are electronically powered wheelchairs, trainers, or adapted ride-on vehicles (Guerette et al., 2013; Livingstone and Field, 2014; Rosen et al., 2018). PMDs allow individuals with motor disabilities to explore and learn about their environment in a safe manner with less effortful demands. However, most existing PMDs are accessed and controlled through methods that require some reliable, functional movement to operate (e.g., mechanical switches, proximity switches or proportional joysticks). These control schemes are not feasible for the most severely disabled children. Although there has been some success in training children with complex physical needs to operate PMDs through dedicated training programs and careful placement of access method (Huang et al., 2014; Kenyon et al., 2015, 2017), traditional technologies still require a degree of physical movement to operate that remains unreliable, fatiguing, frustrating, or otherwise unavailable to those most in need (Kenyon et al., 2017). Accordingly, children with severe physical disabilities are often unable to acquire power mobility skills to a level proficient enough to be eligible for power wheelchair provision (Kenyon et al., 2015).

For children without a reliable alternate access method for power mobility, brain-computer interfaces (BCIs) are a promising emerging solution. BCIs employ the exogenous (e.g., attending to a flashing light stimulus) or endogenous (e.g., mental visualization) manipulation of brain activity to control external applications or devices (Wolpaw et al., 2020). The most common non-invasive brain signal acquisition modality used for BCI is electroencephalography (EEG), which involves recording electrical brain activity from sensors placed on the scalp (Abiri et al., 2019). BCIs are being explored across a spectrum of use including for the restoration, augmentation and enhancement of communication, rehabilitation, environmental control, entertainment and locomotion for individuals with disabilities (Nicolas-Alonso and Gomez-Gil, 2012). Commercial-grade EEG-based BCI systems such as the Emotiv Epoc (*Emotiv, USA)* can help address the BCI translation gap as they offer an affordable and tolerable hardware alternative that can be used in a broader range of environments (Lau-Zhu et al., 2019).

Several frameworks have been proposed, prototyped, and evaluated for BCI-enabled PMD control, varying in the type of BCI control paradigm, classification, and navigational schemes employed (Fernández-Rodríguez et al., 2016; Al-qaysi et al., 2018). One of the most common BCI paradigms used for PMD control are event-related desynchronization/synchronization (ERD/ERS)-based paradigms (Fernández-Rodríguez et al., 2016). These paradigms require the user to actively manipulate their brain activity through imagined movement, mental calculations or other mental/visualization tasks (Fernández-Rodríguez et al., 2016; Al-qaysi et al., 2018). ERD/ERS based methods are popular for PMD control as they allow the user to decide asynchronously when they want to activate the system. Other BCI control methods for PMD control, such as visual-evoked potentials (Wang et al., 2008), require the user to attend to a stimulus delivered by the system before a decision can be interpreted from the EEG.

Navigational control schemes also differ for BCI-enabled PMD control. Some frameworks have utilized a low-level, direct mapping of BCI command with wheelchair direction (e.g., forward, right, left, and reverse) (Fernández-Rodríguez et al., 2016; Al-qaysi et al., 2018). In this fashion, the navigation control would function like pressing a button/switch for each driving command. Others have explored high-level, guidance-based methods where the driver uses BCI to select an end-destination, which is preprogrammed into the wheelchair (Fernández-Rodríguez et al., 2016; Al-qaysi et al., 2018). An example of this is outlined in Tang et al. (2018), who implemented a destination-based navigation scheme with a visually-evoked BCI paradigm using computer vision to dynamically identify target destinations in the nearby environment.

Due to a multitude of factors including intricate BCI hardware and software, high cost of equipment, and the complex lives of individuals with disabilities, BCI systems have been slow to transition out of the lab and into the real-world for end-users (Kübler et al., 2020). Out of 35 BCI-enabled PMD studies reviewed in Fernández-Rodríguez et al. (2016), only 3 involved disabled end-users with lived experience, with only a single individual end-user in each study (Alqasemi and Dubey, 2010; Diez et al., 2013; Lopes et al., 2013). BCI translation for pediatric populations has been limited for many reasons, including the added complexity of the developing brain as well as a lack of existing BCI systems adapted for pediatric users (Mikołajewska and Mikołajewski, 2014; Kinney-Lang et al., 2016; Orlandi et al., 2021). Our research team has recently demonstrated that both typically developing children (Zhang et al., 2019) and children with disabilities (Kelly et al., 2020; Jadavji et al., 2021, 2022) can use simple EEG-based BCI systems.

In an initial proof-of-concept study we demonstrated a BCI-PMD prototype that could be controlled with a commercial-grade BCI system by children with quadriplegic cerebral palsy (Floreani et al., 2021). In the current study, we extend this line of investigation to formally assess the feasibility of such a system for this population to explore independent movement. This work looks to assess the suitability and practicality of setup and calibration procedures: the tolerability of the headset; the ability to use BCI to accomplish standard power mobility tasks; and the willingness of children and families to participate in long-term BCI-PMD research programs.

We hypothesized that children with quadriplegic cerebral palsy would be able to accomplish basic power mobility skills reflective of beginner power mobility users. We also hypothesized that the BCI headset would be tolerated well, and participants would experience mild to moderate workload. The development of power mobility skills for children with complex physical needs can take substantial training and practice (Livingstone and Paleg, 2014). Similarly, learning how to use BCI is also a skill that must be acquired over time (McFarland and Wolpaw, 2018). Through this study, we provide the foundation and justification for future exploration of longitudinal BCI-PMD training and skill acquisition for children with severe physical disabilities.

## Materials and methods

### Participants

Children with severe quadriplegic cerebral palsy were recruited for this pilot study from our clinical BCI research program^1^ via clinician referral within our population-based, tertiary care pediatric hospital (Jadavji et al., 2022). General inclusion criteria for the program and the current study were: (1) severe quadriplegia: Gross Motor Function Classification Score (GMFCS) (Palisano et al., 2008) of 5; Manual Ability Classification Score (MACS) (Eliasson et al., 2006) of 4 or 5, indicating non-ambulatory with minimal to no functional hand use; (2) age 6–18 years; and (3) estimated grade 1 developmental cognitive capacity based on caregiver impressions. Intake screening was performed by a combination of clinical experts to confirm eligibility. Previous experience with BCI and/or power mobility technologies was documented. Assent and parental consent were obtained in accordance with the Conjoint Health Research Ethics Board at the University of Calgary.

### Study overview

Participants attended two BCI power mobility sessions at the Alberta Children’s Hospital (*Calgary, Canada*). A simple, commercial-grade BCI system was used to activate a power mobility trainer device (PMTD). The PMTD is a wheelchair base with an attached platform where children can sit comfortably in their own manual wheelchair, allowing them to explore new access methods without needing a power wheelchair of their own. First, participants were positioned on the PMTD with support from an occupational therapist experienced in seating and power mobility (DR). Then the EEG headset was placed to determine optimal comfort and time to reach satisfactory positioning, signal quality, and time to complete calibration of the BCI system was recorded. Participants then performed two tasks relevant to power mobility driving: (1) controlled activation of the PMTD to assess BCI performance; and (2) directed movement over a specified distance to assess emerging driving skills.

Controlled activation was examined over 10 trials. Participants were asked to engage and maintain a *neutral* (no-activity) state for 5 s to keep the PMTD at rest. After 5 s, they were cued (visually and verbally) to activate the BCI and move the PMTD within 10 s. The distance trials evaluated ability to move the PMTD forward one quarter-length of a gym (∼3.75 meters, on a “low” speed) for 4 trials, followed by moving one half-length of a gym (∼7.5 m, on a “medium” speed) for 2 additional trials. In total, 2 full-length room movements by the BCI were recorded for each participant and session. Endpoints for each trial were marked with playful goals for the participants, including driving an inflatable soccer ball through a net or knocking over a set of bowling pins with the PMTD. Once the endpoint was reached, participants were instructed to return the PMTD to rest by ceasing activation of the BCI. Stopping the PMTD was considered successful if the participant was able to engage their neutral command and cease PMTD motion within 1 m of the target line. If participants moved beyond this line, the PMTD was stopped by one of the researchers via an emergency stop button. Total time taken to travel the specified distance, number of BCI activations produced, and successful stops were recorded for each trial.

### Equipment

#### BCI systems

**FIGURE 1:**
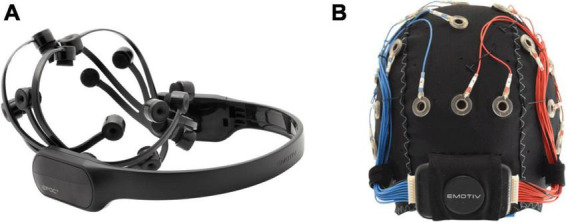
Emotiv BCI headsets. **(A)** Headband-style Emotiv Epoc X with 14 electrodes on flexible plastic arm bands; **(B)** cap-style Emotiv Flex with flexible positioning options for saline-based electrodes.

The Emotiv Epoc X and the Emotiv Flex (*Emotiv, USA)* were used for this study. The Epoc X is a headband-style headset consisting of 14 channels (AF3, F7, F3, FC5, T7, P7, O1, O2, P8, T8, FC6, F4, F8, AF4) with saline-wet sensors attached to flexible plastic arm bands (Figure 1) and sampling rate of 128 Hz. The Emotiv Flex is a cap-style headset with up to 32 electrode leads available for placement at standard 10/20 EEG locations and similar sampling rate. The Emotiv Flex was chosen for participants who exhibited frequent uncontrolled gross motor movements (e.g., dystonic/dyskinetic movements), as the Flex system could be more firmly stabilized on the head. For consistency, 14 electrodes were placed at the same locations as the Epoc X. These commercial systems were chosen due to the tolerability of the saline electrodes (no gel required, higher comfort than available dry comb electrodes) and participant familiarity with the system from their involvement in our clinical BCI program (Jadavji et al., 2022).

**FIGURE 2:**
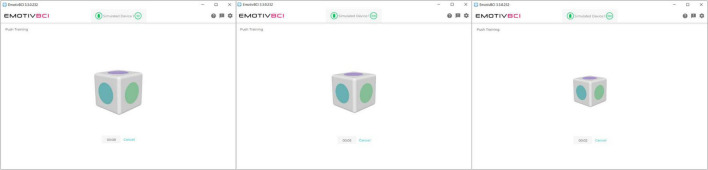
Visual feedback in the Emotiv BCI calibration software. The user executes their imagined mental command to “push” the cube into the screen. The cube shrinks in size, giving it the effect of being pushed back into the screen.

An integrated *mental command* paradigm was used for BCI control. This involved training a mental visualization, in the form of an imagined movement or action, in combination with visual neurofeedback. An example of a commonly-used mental command is *push.* A visual feedback icon in the software is animated to move backward into the screen in response to the strength of the detected “push” command (Figure 2). EEG collected during the active command portion of training is then compared to EEG features collected during a *neutral* or rest state. A classifier is trained to discriminate between the *active* and *rest* states, enabling online and real-time detection between these conditions. Since data are processed and classified through Emotiv’s cloud-based software service, *Cortex*, details on the actual classification scheme are limited. The mental command paradigm is likely more akin to an attention-based paradigm (i.e., assessing changes in activity in the prefrontal cortex during a focused mental state) than an ERD/ERS BCI paradigm (i.e., motor imagery) due to the limited electrodes over the motor cortex region of the scalp (Pfurtscheller and Neuper, 2001).

#### BCI-to-switch interface

**FIGURE 3:**
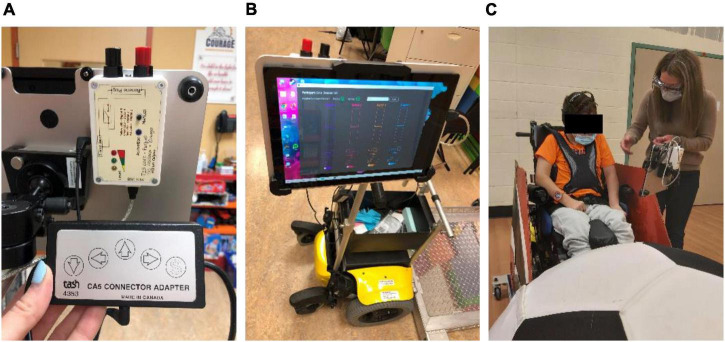
BCI-to-switch interface. **(A)** The BCI-to-switch interface device, connected to the “forward” driving command of the wheelchair switch controls; **(B)** the BCI-to-switch interface custom software, displaying system controls and visual feedback to the user on a tablet computer; **(C)** the BCI-to-switch interface and tablet computer mounted to the PMTD in the participant’s line of sight.

BCI output commands were translated to control of the PMTD through a custom-developed BCI-to-switch interface (Figure 3A). Detected BCI commands were streamed and mapped to the activation of general-purpose input/output pins of a microcontroller through custom software and USB connection. The microcontroller directed the activation of four standard 3.5 mm mono jack outputs. Each mono jack output could be mapped to a different drive command (forward, left, right and reverse) of a PMD with existing switch controls. The custom software included tools to monitor BCI signal quality, adjust system parameters, toggle the BCI control on and off, and display visual feedback to the user (Figure 3B). A Microsoft Surface Pro (*Microsoft, USA*) was mounted on the PMTD within the participants’ line of site so visual feedback could be monitored as they were driving (Figure 3C).

#### Power mobility trainer device

**FIGURE 4:**
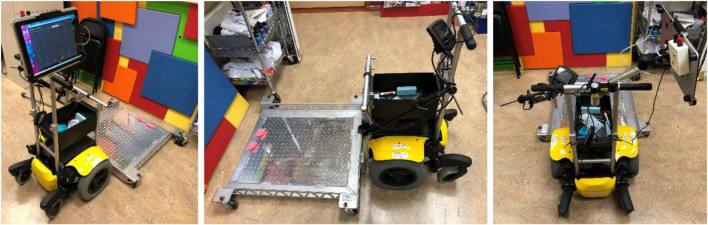
The power mobility trainer device (PMTD), in three different views. The PMTD consists of a power wheelchair base, attached to and propelling a wheeled platform. Participants sit on this platform in their manual wheelchair and can practice using a new access method.

The PMTD used in this study consisted of a wheeled platform attached to and powered by a power wheelchair base (Figure 4). Participants could be secured, seated in their own custom-fit manual chair, on this platform and practice using BCI to move independently. The PMTD could be controlled with standard switch activations for driving forward, left, right and reverse. The speed of the PMTD was preprogrammed to enable 3 speed profiles: slow (2.0 km/h), medium (2.5 km/h) and fast (3.0 km/h). An emergency stop switch was monitored by the researchers for participant safety.

### BCI calibration

During the first session a personalized imagined action was chosen for each participant. The chosen action was relevant and meaningful to each child (e.g., imagining making their manual wheelchair “go,” or imagining “sticking out their tongue”) and was used for both sessions. Cues to encourage the participant stay calm and relaxed for the “neutral” command were also determined with input from the child, caregivers, and clinical team experts. Again, cues were chosen to be meaningful for each child (e.g., counting backward from 10, caregiver singing a calming song). A list of each participant’s commands and cues can be found in Table 1.

**TABLE 1:** Participant mental command and neutral strategies and cues.

Participant	Mental command and cues	Neutral cues
P1	Imagine pushing wheelchair like Spiderman	Thinking about Peter Parker to stay calm/relaxed
P2	Imagine pushing their wheelchair	Caregiver counting backward from 10
P3	Imagine sticking out their tongue	Thinking of nothing, quiet
P4	Imagine puckering lips/giving Mom a kiss	Thinking of the color orange
P5	Imagine making their adapted toy jeep “go”	Caregiver singing “5 little ducks” song
P6	Imagine pushing with their arms	Counting backwards from 10
P7	Imagine making the wheelchair “go”	Caregiver counting backwards from 10
P8	Imagine making the wheelchair “go”	Caregiver counting backwards from 10

**FIGURE 5:**
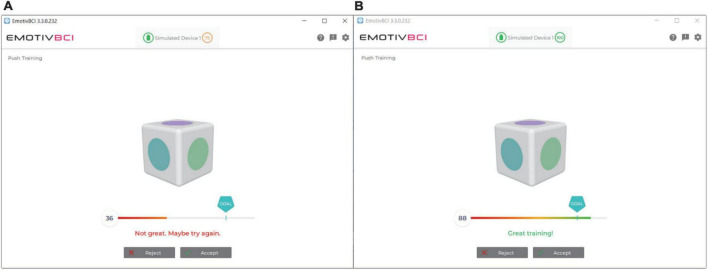
Emotiv BCI App user interface with calibration score feedback. **(A)** Poor calibration trial. Trials with such scores were rejected and re-attempted. **(B)** Great calibration trial. Scores with this rating were kept.

Emotiv’s *BCI App* was used to calibrate the BCI system. A single training trial in the Emotiv BCI App consisted of 8 s of focused mental attention, either performing the desired imagined action for the command state or resting for the neutral state. The command and the neutral state were trained 8 times each; this amount was chosen based on pilot work with the participants in our clinical BCI program. Training trials were scored by the Emotiv BCI App. Trials that were awarded a classification of “not great” or lower were rejected and re-attempted up to three times, at which training was accepted in order to proceed through the session and minimize participant frustration (Figure 5). Trials where the participant was moving excessively were also rejected and re-attempted.

### Post-session questionnaire

After the session was completed participants self-reported, using a 5-point Likert scale, their overall enjoyment of the session. The NASA-TLX, adapted for children (Laurie-Rose et al., 2017), was used to assess workload. The NASA-TLX is a self-assessment of perceived workload across six dimensions – physical, mental, and temporal demand and effort, frustration, and performance. It is a commonly used metric for assessing BCI usability (Kübler et al., 2014; Rhiu et al., 2019). Adaptations to the NASA-TLX for children included: simplifying the language of the questions, using symbolic representations in addition to numerical responses, and reducing the total number of response options (Laurie-Rose et al., 2017). Non-verbal participants used partner-assisted scanning and their communication cue/gesture for “yes” to select their desired rating. Due to fluctuating ability to produce a reliable communication gesture, some participants were not able to answer all questions on each session day. Participants and parents/caregivers were also asked if they would like to participate in future BCI power mobility research studies.

### Data analysis

BCI performance was calculated by tallying correctly executed tasks (e.g., activating the PMTD within 10 s) for the controlled activation trials and dividing by the total number of attempts. Cohen’s kappa, a measure of agreement between target task and the actual task executed by the participant, was also calculated to facilitate comparison of the present results with related BCI literature (Zhang et al., 2019; Jadavji et al., 2021). The kappa score can range from -1 to + 1, with + 1 indicating perfect agreement and 0 representing chance agreement. Driving skills were quantified through aggregated measures of average driving speed, distance per activation, duration of activation, and ability to stop at the target endpoint. Differences in performance between sessions were calculated using a two-tailed paired *t*-test. For the driving tasks, differences between distance length and session were assessed using a two-factor ANOVA. Note that due to the low power of this study, presented results reflect observed trends only.

## Results

### Participation

8 children with severe quadriplegic cerebral palsy were recruited. These represented the first 8 families who were invited (i.e., recruitment rate was 100%). Participants ranged in age from 6 to 16 years (mean 11.3 ± 3.3) and 5 were male. Gender balance was not sought due to multiple factors including young age and complex communication needs.

Participants had a range of previous BCI experience (1 month to 5 years), based on their time within our clinical BCI program. 5 participants had one prior exposure using BCI to drive a PMD when participating in our proof-of-concept study (Floreani et al., 2021). 2 participants (P4 and P6) had previous power mobility experience using alternative access technologies (i.e., switches and/or joysticks), but struggled to use them effectively for functional driving. 1 participant (P1) had limited ability to self-propel a one-arm drive manual wheelchair. 3 participants (P5, P7, P8) had no prior experience with independent mobility. See Table 2 for participant details.

**TABLE 2:** Participant information.

Participant	Age	Sex	Diagnosis^1^	GMFCS^2^	MACS^3^	BCI exp	Mobility exp
P1	10	M	QCP	5	4	2 years	Manual, limited
P2	9	M	QCP	5	5	2.5 years	BCI, once^4^
P3	14	M	QCP	5	5	2.5 years	BCI, once^4^
P4	14	M	QCP	5	5	4–5 years	Mouth-operated joystick, limited
P5	6	F	QCP	5	4	1 month	None
P6	16	F	QCP	5	5	3–4 years	Head array, limited
P7	12	M	QCP	5	5	6 months	None
P8	9	F	QCP	5	5	3 months	None

^1^QCP, Quadriplegic cerebral palsy; ^2^GMFCS, Gross Motor Function Classification Score (Palisano et al., 2008); ^3^MACS, Manual Ability Classification Score (Eliasson et al., 2006); ^4^Participants used BCI to activate a PMD in Floreani et al. (2021).

Six of the eight recruited participants completed both study sessions. P7 was unable to complete their second session due to the onset of tightening COVID-19 lockdown measures. Their results from session 1 are presented but are excluded from group statistical analyses. P8 did not finish all distance trials in their second session due to fatigue and declining mood after a long day of school. All participants and families expressed interest in trying BCI-power mobility again in the future.

### Setup and calibration

Total time needed to don the headset, achieve a suitable EEG signal quality (>67% “contact quality,” measured using the Emotiv BCI App), and calibration is presented in Table 3. Setup time took an average of 7.72 ± 3.7 min. The biggest factor impacting setup time was participant hair length/thickness, where long, thick hair (e.g., P6) required substantial additional time as compared to short, thin hair. Calibration time took an average of 12.39 ± 3.9 min. Issues that increased calibration time included unavailable proper size of headset/cap (e.g., P5 S1, P8 S1), distractions from caregiver or other individuals in the room during the session (e.g., P8 S2), and poor participant mood (e.g., P8 S2). For P7, both setup and calibration took longer as the participant had cochlear implants, which required slightly more complex positioning under the EEG cap.

**TABLE 3:** Brain-computer interface setup and calibration times.

Participant	Session	Setup time (min)	Calibration time (min)
P1	S1	12.00	8.05
	S2	7.93	11.05
P2	S1	9.23	13.00
	S2	5.70	9.08
P3	S1	8.28	12.97
	S2	5.05	9.17
P4	S1	3.00	12.32
	S2	4.03	11.33
P5	S1	6.25	16.97
	S2	10.00	8.90
P6	S1	12.92	8.50
	S2	5.12	9.42
P7	S1	14.67	19.90
	S2	–	–
P8	S1	9.50	18.00
	S2	2.10	17.23
Average		7.72 ± 3.7	12.39 ± 3.9

### Headset tolerability and enjoyment

All participants reported the BCI headsets as tolerable for the duration of both sessions. There were no reports of discomfort or irritation caused by the headsets. On average, participants rated their enjoyment of using BCI for driving as a 4.5 out of 5 (for participants able to provide a numerical ranking). The 2 participants who used binary selections chose “I liked driving with BCI” over “I did not like driving with BCI.”

### BCI performance

**FIGURE 6:**
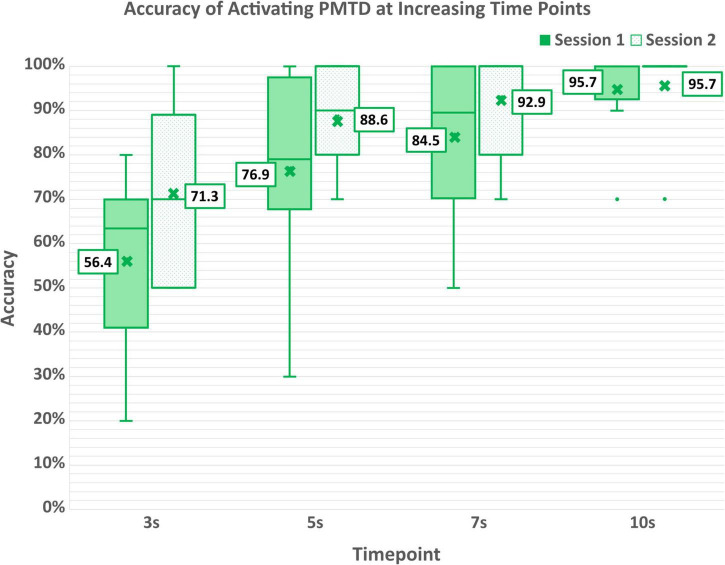
Accuracy of activating the PMTD within increasing time windows of 3, 5, 7, and 10 s.

Participants were able to effectively engage their active mental command to initiate movement of the PMTD within 10 s after cueing, achieving an accuracy of 95.3 ± 10.6% (95.0 ± 10.7% for session 1; 95.7 ± 11.3% for session 2), as shown in Table 4A. Six of the eight participants were able to activate the PMTD within the 10 s for every single trial. P2 was unable to activate the PMTD three times in each of their sessions while P7 was unable to activate the PMTD only once. Accuracy of activating the PMTD within shorter time windows (3, 5, and 7 s) are shown in Figure 6. Participants activated the PMTD within 3 s of the cue 63.3 ± 20.5% of the time, within 5 s 82.3 ± 18.9% of the time, within 7 s 88.4 ± 15.8% of the time.

**TABLE 4:** Accuracy of (A) activating the power mobility trainer device (PMTD) within 10 s of cueing, (B) holding the PMTD still for 5 s after cueing, (C) total accuracy across both activating and holding still, (D) kappa score across both activating and holding still.

Participant	Session	(A) Accuracy of activation (within 10 s)	(B) Accuracy of holding still (for 5 s)	(C) Total accuracy	(D) Cohen’s kappa
P1	S1	100.0%	60.0%	75.0%	0.6
	S2	100.0%	50.0%	75.0%	0.5
P2	S1	70.0%	80.0%	75.0%	0.5
	S2	70.0%	70.0%	70.0%	0.4
P3	S1	100.0%	100.0%	100.0%	1
	S2	100.0%	50.0%	75.0%	0.7
P4	S1	100.0%	100.0%	100.0%	1.0
	S2	100.0%	70.0%	85.0%	0.7
P5	S1	100.0%	30.0%	65.0%	0.3
	S2	100.0%	40.0%	70.0%	0.4
P6	S1	100.0%	70.0%	85.0%	0.7
	S2	100.0%	90.0%	95.0%	0.9
P7	S1	90.0%	50.0%	70.0%	0.4
	S2	–	–	–	–
P8	S1	100.0%	50.0%	74.0%	0.49
	S2	100.0%	22.0%	61.0%	0.22
Average	S1	95.0 ± 10.7%	67.5 ± 24.9%	80.5 ± 13.3%	0.62 ± 0.26
	S2	95.7 ± 11.3%	56.0 ± 22.5%	75.9 ± 11.1%	0.52 ± 0.22
	Average	95.3 ± 10.6%	62.1 ± 23.7%	78.3 ± 12.1%	0.57 ± 0.24

Participants had more difficulty engaging their neutral strategy to hold the PMTD still. Participants were able to successfully hold the PMTD still in 62.1 ± 23.7% of trials (67.5 ± 24.9% for session 1; 56.0 ± 22.5% for session 2), as shown in Table 4B. Greater variability in participant ability to hold the PMTD still was observed. When early/accidental activations occurred, there was an average of 1.2 ± 0.3 early activations per trial, with an average onset of 2.04 ± 0.55 s and an average duration of 1.43 ± 0.42 s.

Examining the joint tasks of holding the PMTD still then activating on cue resulted in a total average classification accuracy of 78.3 ± 12.1% across the two sessions (80.5 ± 13.3% for session 1; 75.9, ± 11.1% for session 2). Average Cohen’s kappa score was found to be 0.57 ± 0.24 (0.62 ± 0.26 for session 1; 0.52 ± 0.22 for session 2). Total average classification accuracies and kappa scores can be found in Table 4C,D. No statistically significant differences were found across sessions for any measures of performance.

### Power mobility skills

#### Distance trials

**FIGURE 7:**
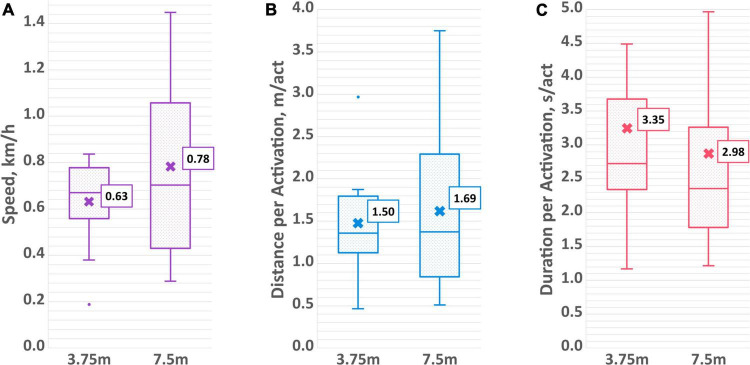
Aggregated driving measures of **(A)** average speed, **(B)** average distance per activation, and **(C)** average duration per activation for both the 3.75 and 7.5 m distance trials.

On average over both sessions, it took participants 33.1 ± 24.7 s to move the PMTD forward 3.75 meters and 49.9 ± 31.3 s to move 7.5 meters. Substantial variability across participants was observed, with some averaging as low as 17 s to traverse the 3.75-meter distance, with others requiring on average upwards of 100 s (range 17.1–134.9 s). Time and distance were aggregated into average speed (expressed in km/h). Participants traveled at an average speed of 0.63 ± 0.18 km/h for the 3.75 m distance and at an average speed of 0.78 ± 0.38 km/h for the 7.5 m distance. Average speed is displayed in Figure 7A, while raw times taken to complete the distance trials can be found in the Supplementary material.

Number of individual activations to advance the PMTD the specified distance was also recorded. For the shorter distance, participants activated the PMTD an average of 4.1 ± 1.6 times, while for the longer distance the average was 7.0 ± 3.7 times. This works out to be an average of 1.50 ± 0.57 and 1.69 ± 1.07 meters per activation for the short and long distances, respectively. On average, the duration of an activation was 3.35 ± 1.99 s for the 3.75 m distance and 2.98 ± 1.60 s for the 7.5 m distance. The aggregated measures of average distance per activation and duration per activation can be found in Figures 7B,C while the average number of activations can be found in the Supplementary material. No statistically significant differences between sessions or distance length were found for any of the measures of driving skills.

#### Stopping

**FIGURE 8:**
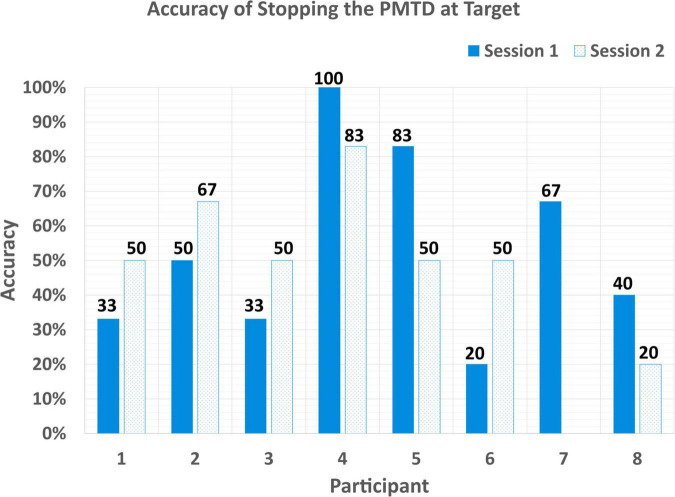
Accuracy of successfully stopping the PMTD within 1 m of a target.

Most participants had difficulty stopping the PMTD on cue. Participants stopped the PMTD at the target only 53.1 ± 23.3% of the time (53.3 ± 27.7% for session 1; 52.9 ± 19.2% for session 2), as shown in Figure 8. Again, variability can be seen here across participants, with stopping accuracy ranging from 100% (P4 S1) to 20% (P6 S1).

### Previous BCI experience and age

**FIGURE 9:**
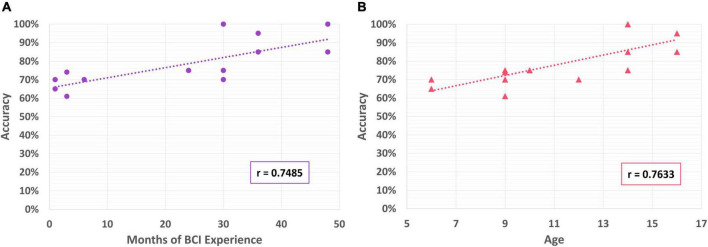
**(A)** Correlation of BCI performance with months of BCI experience; **(B)** correlation of BCI performance with participant age.

Participants varied in the amount of previous experience they had with BCI technologies. Some had been involved in our clinical BCI program for only a few months, others for several years before taking part in the current study (Jadavji et al., 2022). Previous BCI experience was estimated by the number of months they had participated in the BCI program. Amount of prior BCI experience was found to be positively correlated with accuracy in controlling the PMTD (*r* = 0.749), see Figure 9A. Age was also found to be positively correlated with control accuracy (*r* = 0.763), see Figure 9B. There was no correlation found with BCI experience or age and any of the aggregated driving skill measures (speed, distance/activation, duration/activation).

### Workload

**FIGURE 10:**
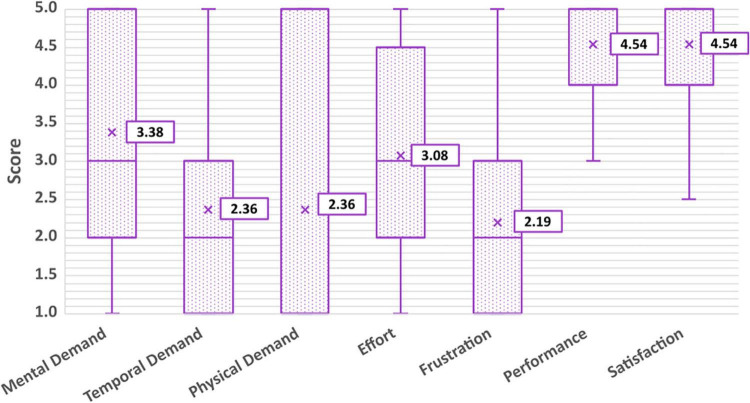
Average workload scores according to the six dimensions of the NASA-TLX, and overall satisfaction.

Average scores for each dimension can be found in Figure 10. Lower scores are associated with a lower experience of that dimension. For example, a “1” on the frustration Likert scale corresponded to “not at all frustrated” while a “5” corresponded to “very frustrated,” and a “1” on the performance scale corresponded to “not proud at all” while a “5” corresponded to “very proud.” Scores for individual dimensions can be found in the Supplementary material. On average, participants found that driving the PMTD with BCI required a moderate amount of mental demand (having to do lots of thinking to complete the tasks; 3.38 ± 1.39) and effort (3.08 ± 1.44) but a low to moderate temporal (feeling rushed or hurried to complete the tasks; 2.36 ± 1.36) and physical demand (having to expend lots of physical energy to complete the tasks; 2.36 ± 1.80). Participants experienced a low to moderate amount of frustration (2.19 ± 1.35), were proud of their performance (4.54 ± 0.88) and had high satisfaction with their experience with BCI driving (4.54 ± 0.80). No significant effect on BCI performance or driving skills were found for any of the workload dimensions. All participants and families reported wanting to try BCI-enabled power mobility again in the future, including participating in future research studies.

## Discussion

This pilot study looked to assess the feasibility of BCI-controlled access to power mobility for children with severe physical disabilities using a simple commercial-grade BCI headset. Overall feasibility was confirmed, including the practicality of setup and calibration procedures, tolerability of the headset, accuracy of control, ability to execute basic power mobility skills, workload, and enjoyment of using the BCI system. This work provides the foundation required to optimize the design of the longitudinal studies for realizing the possibility of BCI power mobility for severely disabled children.

### Practicality of setup and calibration procedures

The time needed to set up and calibrate equipment is a significant factor that can affect the usability of BCI as an access method. Long or complex setup procedures can cause frustration and lead to device abandonment (Petrie et al., 2018), particularly for children with complex needs. In this study, setup took an average of 7.72 ± 3.7 min and calibration took an average of 12.39 ± 3.9 min, with headset fit and hair length as the primary factors affecting these times. Participants who had longer hair required more time to work sensors through the hair to achieve sufficient contact quality and required more frequent rehydration of the saline-based sensors, both of which contribute to the setup and calibration time. Participants with longer hair may benefit from dry, comb-based electrodes that can effectively work through hair to reach the scalp.

The choice to use a commercial-grade, saline-based headset in this pilot study was to help reduce the burden of setup with less technical positioning, lower density of sensors, and eliminate the need for application and corresponding clean-up of electrode gel. The use of electrode gel has previously been reported as frustrating to end-users (Holz et al., 2015). Other researchers, like Al-Taleb et al. (2019) who used the Emotiv Epoc headset in their study found comparable setup times to what we have reported (78% of participants took within 5–10 min to set up, the remaining 22% took 10–15 min). The benefit of reduction in setup time offered by commercial-grade headsets may, however, come at a cost of decreased signal quality and adjustability of headset fit (Duvinage et al., 2013). Of the two commercial headsets used in this study, it was found that the increased adjustability to different head sizes and shapes offered by the cap-style Emotiv Flex was critical for participants who struggled with reliable positioning and sensor contact with the Epoc X system. Further work should prioritize the design of low-cost, flexible and easy-to-apply BCI hardware suitable for both pediatric and adult end-users.

### Tolerability and workload

Overall, participants tolerated the BCI headset and driving activities very well. There were no reports of discomfort or irritation from the headsets, and they were able to wear the headset for the full duration of both sessions. Only one participant did not complete a session, but this was attributed to their overall mood on the session day rather than the BCI headset or driving activities. Similar tolerability of the Emotiv Epoc system has been previously reported in Al-Taleb et al. (2019), where <10% described the device as uncomfortable, and none experienced irritation from the saline solution.

Participant reports of moderate workload in the domains of *mental demand* and *effort* and low-moderate workload in the domains of *temporal demand, physical demand*, and *frustration* are in line with expected workload results from previously published BCI usability studies. These have reported moderate workload for a range of BCI paradigms including P300-based paradigms and SMR (motor imagery)-based paradigms (Holz et al., 2013; Zickler et al., 2013; Daly et al., 2014; Kübler et al., 2014). Botrel et al. (2015) similarly found participants rated mental workload the highest out of all workload dimensions, attributing this to the high degree of concentration required for execution of BCI tasks. Given the attention-dependent nature of the mental command activation used in this study, it is not surprising that higher scores in mental demand and effort were reported. No previous usability studies have investigated how workload changes over long-term use of BCI. Investigations into how continued, long-term use and practice of BCI affects reports of workload are of potential interest for future work, as this would affect feasibility of BCI as a long-term solution for functional power mobility use. It would also be of interest to compare experiences of workload using access methods relying on physical function (i.e., mechanical switches) to experiences of workload using BCI for children who are able to exhibit some (yet unreliable) voluntary motor control, to formally assess if BCI could be a less demanding access solution for them. The reported high satisfaction in using BCI for movement exploration suggests participants found this to be a worthwhile and rewarding activity.

### BCI performance

Our observed results reveal several potential insights for continued BCI-enabled power mobility investigations in children with severe physical disabilities. First, the findings suggest that it may have been easier for participants to activate the PMTD compared to holding it still. We suspect part of this may be due to the excitement and anticipation of the upcoming movement, and the desire to activate the BCI to begin the activity. Engaging and maintaining the neutral state requires the participant to remain relaxed, calm the body (as much as possible) and clear the mind. Each of these self-regulatory behaviors may be a challenging task for children in general given that networks related to self-regulation and effortful control continue to develop throughout childhood (Posner and Rothbart, 2000, 2009). This may be further accentuated in children with complex physical needs. Future work should evaluate measures of participant temperament, particularly effortful control, as well as the impact of self-regulation strategies on BCI performance in a mental command-based paradigm for power mobility. There is some emerging evidence that behavioral strategies such as mindfulness practice can improve EEG-based BCI task performance (Tan et al., 2014; Stieger et al., 2021). Similar approaches could be investigated and validated for children with disabilities, again in the context of BCI for power mobility control.

BCI performance reported in this study are similar to previously published results evaluating competent use of BCI systems by both typically developing children (Zhang et al., 2019) and children who experienced perinatal stroke (Jadavji et al., 2021). Zhang et al. (2019) reported an average kappa score of 0.46 (range 0.025–0.90) with typically developing children using the Emotiv Epoc headset, similar to our findings here for children with quadriplegic cerebral palsy (mean 0.57, range 0.22–1.00). Given that a score of 0.4 or greater has been used as a cut-off competency threshold in adult BCI literature (Friedrich et al., 2012), this study demonstrates that the majority of children who participated were able to competently use the BCI system as well as their typically developing pediatric and adult peers (13 of the 15 sessions met the competency threshold).

The finding that BCI performance correlated with both prior BCI experience (*r* = 0.75), and participant age (*r* = 0.76) was not unexpected. BCI control is well-known to be a skill that must be developed with continued experience and practice (McFarland and Wolpaw, 2018). In addition, as the brain continues to develop throughout childhood, regions like the prefrontal cortex that regulate attention and focus are still actively developing and not yet fully mature until adulthood, making it likely that BCI performance will improve with age (Lenroot and Giedd, 2006; Tsujimoto, 2008; Orlandi et al., 2021). In Zhang et al. (2019), a significant correlation of BCI performance was also found with age, but this was not replicated in Jadavji et al. (2021). In both, participants were naïve to BCI so the impact of BCI experience could not be explored, but could suggest why participants in the current study exhibited slightly better performance. Further investigation is needed to substantiate these claims and better elucidate the effects of both age/developmental stage and prior experience on BCI performance, in neurotypical and neuro-atypical children.

### Power mobility skills

Field and Livingstone (2018) describe three stages of learning in power mobility skill development: exploratory; operational; and functional. Exploratory learners are focused on building an awareness of cause and effect – that their actions generate movement of the power mobility device. Operational learners are focused on developing the basic skills required to maneuver the power mobility device, while functional learners are progressing toward driving effectively and safely in a variety of environments. The 3PM is a tool designed by Gefen and Rosenberg (2022) to assess power mobility skill development within these three categories of learning. Although not directly applicable to BCI, it has been used in the current study to estimate where participants fall along this continuum of learners.

In our proof-of concept study (Floreani et al., 2021), we found that participants achieved several of the basic skills of exploratory learners (Field and Livingstone, 2018), including demonstration of “awareness of motion,” “cause and effect,” “initiates motion” and “emotional response to motion.” In the present study, findings indicate the participants would be classified as beginner “operational” learners (Gefen and Rosenberg, 2022). Participants demonstrated that key operational skills were emerging as on average, participants were able to move the PMTD using BCI for ∼1.5 meters continuously (1.50 ± 0.57 m/activation) or continuous durations of ∼3 s (3.35 ± 1.99 s/activation), and were able to stop at a target 53.1 ± 23.3% of the time. Interestingly, no significant differences were found between activation length or duration for the short distance compared to the long distance, indicating that these skills could be sustained throughout a longer period of driving time. To become proficient operational learners, participants must be able to drive continuously greater than 3 meters at a time, drive for longer (>30 s) periods of time (although not necessarily in a single activation) and exhibit control over stopping (Gefen and Rosenberg, 2022). Some skills indicated in the 3PM reflective of operational learners, such as “turns right or left” or “reverse,” were not able to be assessed as additional BCI commands must be identified, trained, and evaluated in order for participants to execute these actions. The identification and mapping of additional BCI commands for power mobility control must be explored in future work.

Progression to initial stages of the “operational learner” category within two BCI power mobility training sessions is positive evidence for the feasibility of BCI as an access method for control for this population. According to Field and Livingstone (2018), what truly categorizes a power mobility learner is the speed at which they progress through the different learning stages. Children with less severe disabilities who are able to use access methods such as proportional joysticks effectively may progress through the stages and reach the “functional” level quickly, while others with multiple, complex disabilities who require alternate access methods like mechanical switches or head arrays (proximity switches) may take longer to reach this stage (Livingstone and Field, 2020). Children who may remain indefinitely at the “exploratory” or “operational” stage can still greatly benefit from power mobility training and experience (Livingstone and Paleg, 2014). Further, longitudinal training and investigation of BCI-enabled power mobility will be needed to truly assess what stage of power mobility proficiency children with severe physical disabilities will be able to achieve using BCI, as both power mobility (Livingstone and Field, 2020) and BCI (McFarland and Wolpaw, 2018) involve skills that must be developed over time. Tailored interventions and dedicated training programs can be used to facilitate learning, especially in the case of children with complex physical needs (Kenyon et al., 2015, 2017, 2021).

## Conclusion

In this study, we established the feasibility of using a simple BCI system to enable children with severe physical disabilities to access movement using a power mobility training device. Setup and calibration procedures were found to be practical, provided that the headset fit suitably and could be stably positioned on the participant’s head. Participants tolerated the BCI headset and driving activities well, experiencing only low to moderate workload, low frustration, high satisfaction, and high pride in their performance. Total average BCI control accuracy was similar to prior pediatric BCI studies and surpassed frequently cited cut-off thresholds of efficacy used in adult BCI studies. Participants were more reliably able to activate their mental command (and the PMTD) on cue than sustain their neutral state on cue and were more successful at moving the PMTD compared to stopping. Basic driving skills were assessed, and participants fell in the category of “emerging operational learner,” which is understandable for their lack of prior exposure to power mobility and use of a new, alternative access method (BCI). All participants and families were interested in participating in future BCI-power mobility research and activities. Longitudinal investigations of power mobility and BCI skill training and development will be crucial for determining the degree to which BCI can be used to enable children with physical disabilities access to independent movement. Whether learners progress to the functional “driving” stage using BCI, or remain at the exploratory or operational stages, access to independent mobility and movement can be beneficial to this population to increase their participation, sense of independence and autonomy, and overall quality of life.

## Data availability statement

The raw data supporting the conclusions of this article will be made available by the authors, without undue reservation.

## Ethics statement

The studies involving human participants were reviewed and approved by University of Calgary Research Ethics Board. Written informed consent to participate in this study was provided by the participants’ legal guardian. Written informed consent was obtained from the individual(s) for the publication of any identifiable images or data included in this article.

## Author contributions

EF designed the study, collected the data, conducted analyses, drafted, and revised the manuscript. DR and DK assisted with recruitment, study design and data collection, and revised the manuscript. EK-L and AK oversaw study design, data analyses, reviewed, and revised the manuscript. All authors contributed to the article and approved the submitted version.

## Footnotes

1
www.bci4kids.com
