# Supplementary figures and images for: On the feasibility of simple brain-computer interface systems for enabling children with severe physical disabilities to explore independent movement
Source: Front Hum Neurosci. 2022 Oct 21;16:1007199. doi: 10.3389/fnhum.2022.1007199 (PMC9633669; doi:10.3389/fnhum.2022.1007199)

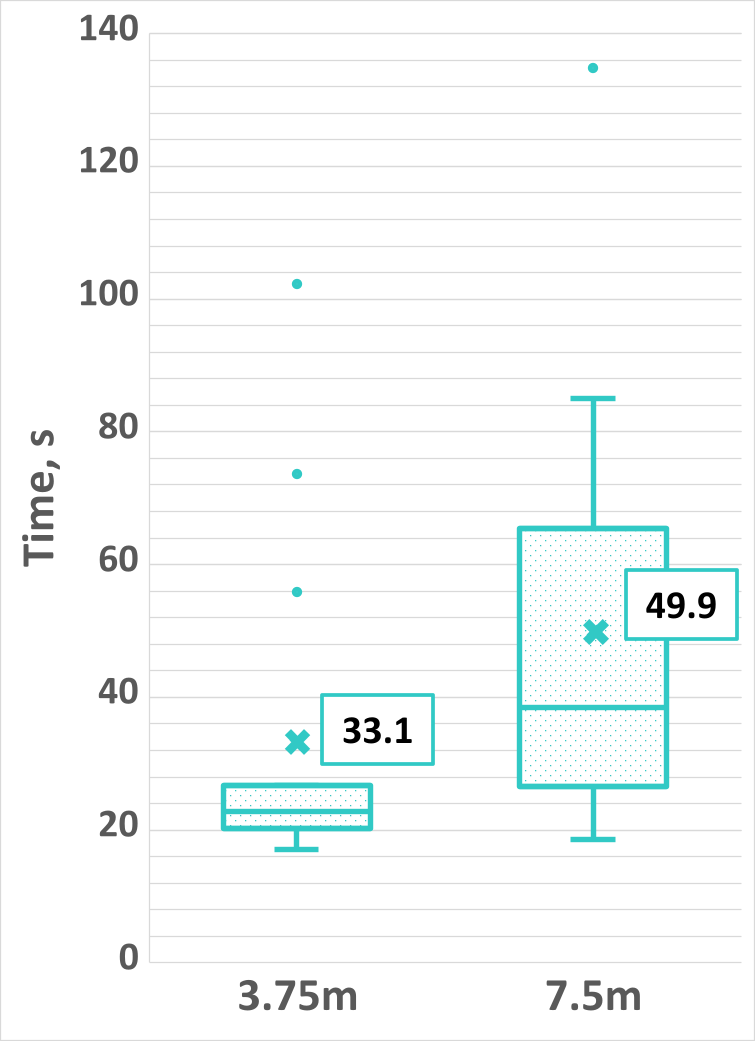

Supplement: Supplementary file 1 [file Image_1.JPEG]

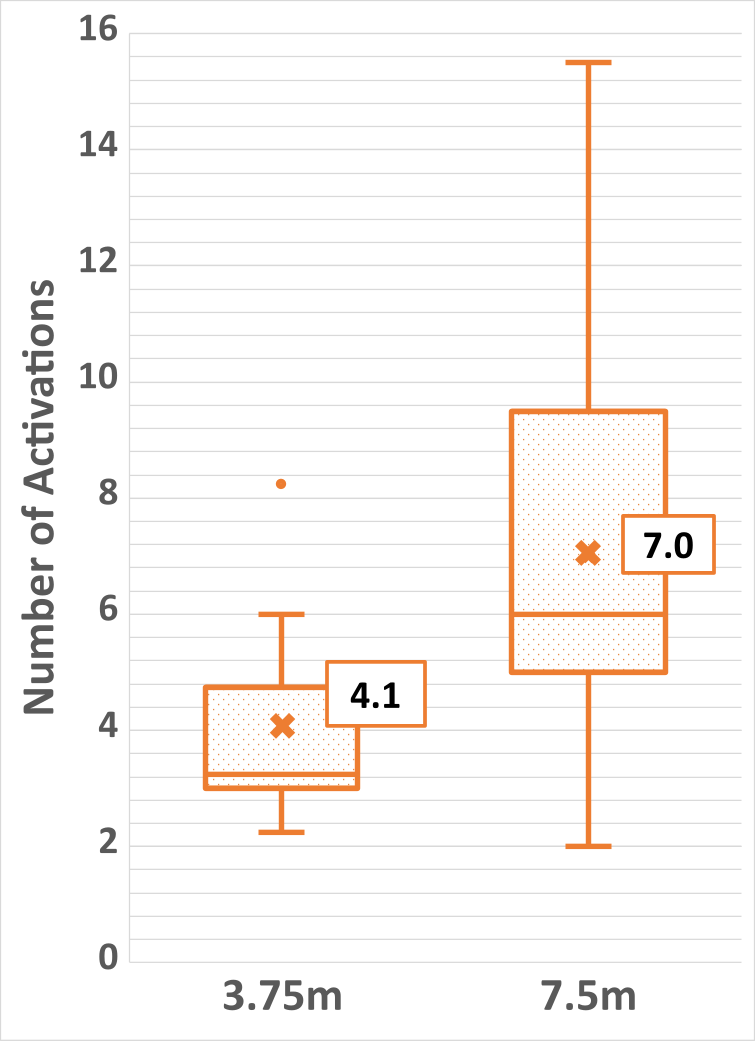

Supplement: Supplementary file 2 [file Image_2.JPEG]

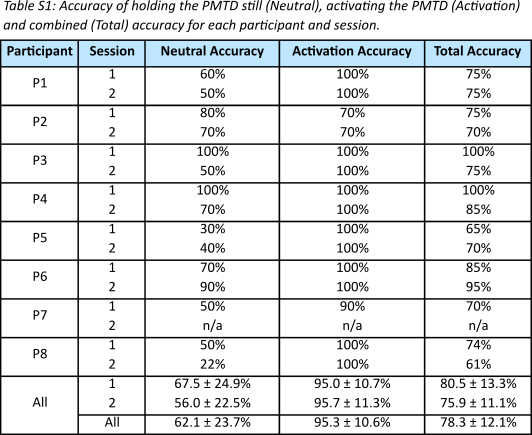

Supplement: Supplementary file 3 [file Image_3.JPEG]

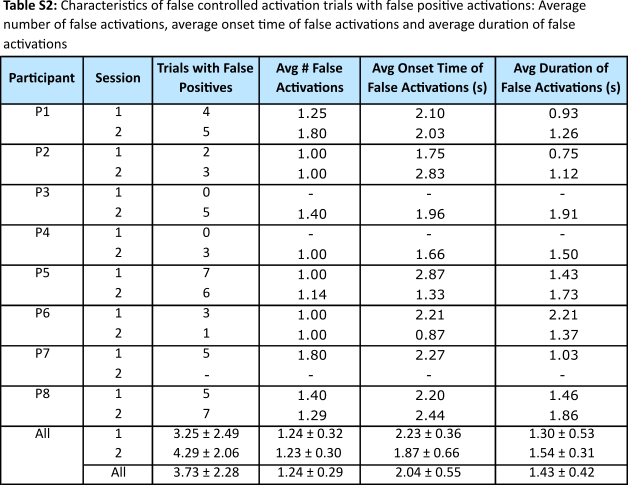

Supplement: Supplementary file 4 [file Image_4.JPEG]

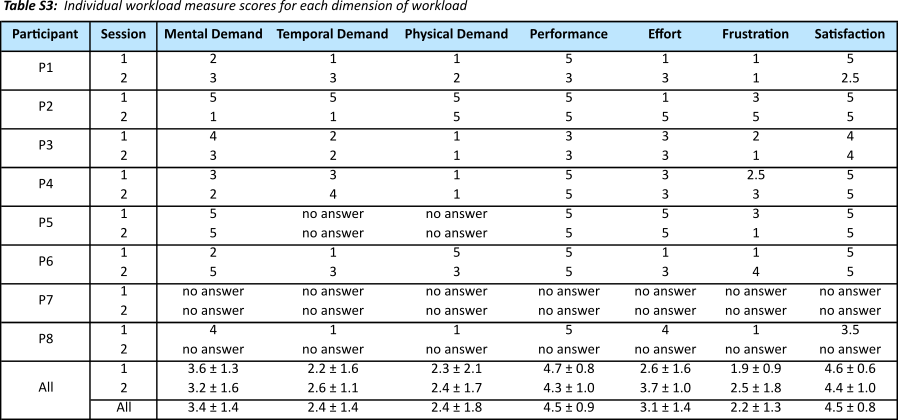

Supplement: Supplementary file 5 [file Image_5.JPEG]
